# Relationship between ADAMTS14/rs4747096 gene polymorphism and knee osteoarthritis in Chinese population

**DOI:** 10.1042/BSR20181413

**Published:** 2018-10-23

**Authors:** Sen Ma, Cheng Ouyang, Shuxin Ren

**Affiliations:** 1 Department of Orthopedics, The People’s Hospital of Nanzhao County, Nanyang City, Henan Province 474650, China; 2 Department of Orthopedics, The Center Hospital of Nanyang City, Nanyang City, Henan Province 473009, China

**Keywords:** ADAMTS14, gene polymorphism, Hardy-Weinberg, knee osteoarthritis

## Abstract

To investigate the association between single nucleotide polymorphisms (SNPs) of A disintegrin and metalloproteinase with thrombospondin motifs (ADAMTS) 14 (*ADAMTS14*) gene and susceptibility to knee osteoarthritis (KOA) in Chinese Han population. Using a case–control design, we enrolled 346 KOA patients and 480 healthy controls. Peripheral blood samples were extracted from each subject. Genotype was determined by sequencing PCR products. The genotype frequencies between cases and controls were compared. The genotype distribution was in accordance with Hardy–Weinberg equilibrium. The minor G allele in case group was significantly higher than in the control group (21.4 compared with 8.8%, *P*=0.000, odds ratio (OR) = 1.71 (95% confidence interval (CI): 1.39–2.11). The GG genotype and the GG/AG combination were more common in the osteoarthritis (OA) group than in the control group. Compared with AA genotype, the GG (OR = 3.09, 95%CI: 2.01–4.75), AG (OR = 2.55, 95%CI: 1.64–3.96), and GG/AG (OR = 1.57, 95%CI: 1.19–2.07) increased the risk of OA. Multiple logistic confirmed the findings by adjusting some potential factors. Subgroup analysis indicated that the ras4747096 was still significantly associated with KOA. There were no significant differences in allele frequency or genotypes frequency for erythrocyte sedimentation rate and C-reaction protein in OA patients (*P*>0.05). ADAMTS14 gene polymorphism was associated with KOA, and the GG genotype increased the risk of KOA in Chinese Han population. The ADAMTS14 may be a diagnostic marker and therapeutic target for KOA treatment. The future study should explore the specific molecular mechanism.

## Introduction

Knee osteoarthritis (KOA) is a serious rheumatic disease characterized by articular cartilage damage and joint space narrowing. In severe cases, when the knee completely loses cartilage, bone and soft tissue structure around the joint will start to change, which could lead to joint pain, swelling, and disability [1,2]. In the United States, symptomatic KOA occurs in approximately 10% of adults aged 60 or above. According to recent statistics, there are 9.3 million adults who are suffering from osteoarthritis (OA) of the knee in the United States. The overall number of KOA is as high as 350 million [3,4]. One in every six persons is suffering from the disease in Asia. With the development in ageing, KOA patients has increased every year, and estimated at approximately 200 million. The ratio of male to female is 1:2. As a result of the ageing process, the number of people with OA of the knee is expected to increase in the coming decades [5]. Pathogenesis of OA has not yet been fully elucidated up to now. It was widely acknowledged that that OA is related to gene and environment factors [6]. It was suggested that the gene accounted for more than 60% of diseases [7]. The human leukocyte antigen class II was the best-known gene in OA, accounting for approximately one-third of OA susceptibility [8]. More gene factors were waiting to be found.

The single nucleotide polymorphism (SNP) is more than 90% of human gene polymorphisms, which is the most common and stable gene variant in the human DNA strands [9]. The coding SNP consisted of synonymous coding SNP and non-synonymous coding SNP. The A disintegrin and metalloproteinase with thrombospondin motifs (ADAMTS) 14 (ADAMTS14) gene rs4747096 is a non-synonymous coding SNP, located on chromosome 10q22.1 [10]. This SNP can change the sequence of amino acid and affects the biological properties. It was known that the overloading, abnormal expression of protease and cytokines, and other factors could lead to imbalance of extracellular matrix decomposition and synthesis metabolism of cartilage, and finally lead to cartilage destruction [11]. ADAMTS play an important role in the degradation of cartilage extracellular matrix [12]. Colige et al. [13] reported that ADAMTS14 was involved in the formation process of collagen fiber II that was one of main components of joint cartilage extracellular matrix. The abnormal metabolism of collagen fiber II decreased the mechanical strength of joint cartilage while this process was one of the important factors affecting joint arthritis. Rodriguez-Lopez et al. [14] found that the G allele in rs4747096 was associated with symptomatic hand OA in Caucasian women requiring total knee replacement because of OA. Currently, there is no report about the relationship between ADAMTS14 gene rs4747096 and KOA. The present study investigates the association between SNPs of ADAMTS14 gene rs4747096 and KOA in Chinese Han population.

**Figure 1:**
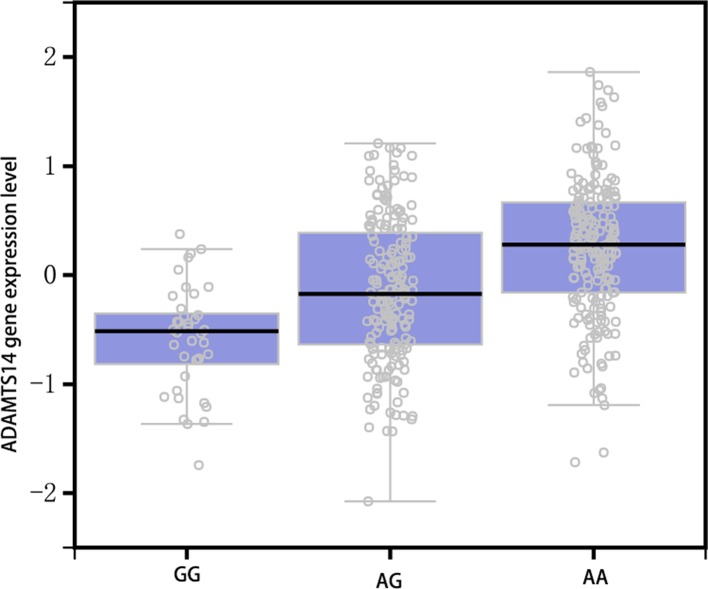
The GTEx dataset revealed a significant eQTL association between the rs4747096 genotype and expression of the ADAMTS14 in peripheral whole blood cells

## Materials and methods

### Study population

We enrolled 346 KOA patients who were diagnosed from 2013 to 2017 in the People’s Hospital of Nanzhao County and the Center Hospital of Nanyang City. The diagnostic criteria of KOA were in accordance with the 2010 American College of Rheumatology/European League against Rheumatism criteria [15]. Patients with severe cardiovascular diseases, severe liver and kidney dysfunction, malignant tumor, and other autoimmune diseases were excluded. Grades 3–4 of radiographic signs of OA according to the Kellgren–Lawrence grading system [16]. The healthy control group was from outpatients of the two hospitals above without the history of OA and autoimmune diseases during the same period. The sample size was calculated using Quanto 1.2.4 and followed by the conditions: α = 0.05, β = 0.10, expected odds ratio (OR) = 1.8, the calculated sample size was 248 in the case group and control group, respectively. The present sample size was enough. We have added this description in the methods. The present study was approved by the institutional review board of Center Hospital of Nanyang City. Written informed consent was received from all study subjects.

### Sample collection and DNA extraction

The general data of case group and two group were obtained via the questionnaire, which was conducted by the trained investigators. The general data included age, gender, smoking, body mass index (BMI), and drinking. Anti-cyclic citrullinated peptide antibodies (ACCP) > 5 RU/ml, antiperinuclear factor (APF) > 5 cells under the microscope, RF (rheumatoid factor) > 20 were positive, and erythrocyte sedimentation rate (ESR) ≥ 20 and C-reactive protein (CRP) > 8 were judged as abnormal [17].

The 5-ml peripheral vein blood was extracted from each study subject. The blood samples were put in EDTA anticoagulant tubes, stored in the refrigerator at −80°C until use. The genomic DNA extraction was completed via Blood DNA Master Kit (QIAamp DNA Blood Mini Kits, Qiagen, Germany). The extracted DNA was dissolved in TE buffer (10 mM Tris, 1 mM EDTA; pH = 7.8), quantitated by measuring the absorbance at 260 nm, and then stored at −20°C for genotyping.

### SNP genotyping

The primer was designed by Generunner 6.2.07 Beta software and synthesized by Beijing Liuhe Huada Gene Company. The genotype was completed by the PCR-restriction fragment length polymorphism (PCR-RFLP). The forward primer sequence: 5′-TGTGCAGGACCAACGCCAACAG-3′ and the reverse primer sequence: 5′-GGAATTGCAGGTAACGGCTCATG-3′. The reaction system included 12 μl 2× Es Taq MasterMix, 2 μl forward and reverse and primer with 10 μl mol/l, 2 μl DNA templates. The PCR program consisted of the following steps: initial denaturation step at 94°C for 2 min, followed by 30 cycles: denaturation at 98°C for 10 s, annealing at 61°C for 30 s, extension at 72°C for 30 s, and extension again at 72°C for 2 min, and stored at 4°C. The PCR product was incubated overnight at 37°C with restriction enzyme. Then the mixtures were electrophoresed and visualized. The expected fragment was 196 bp in AA, 196, 158, and 38 bp in AG, and 158 and 38 bp in GG.

### Statistical analysis

The continuous data were expressed using means ± S.D., and the comparison between case and control groups was performed by Student’s *t* test. The categorical data were expressed using count and percent, and Chi-square test was applied. The Hardy–Weinberg equilibrium test was conducted in the case and control groups. The risk of gene for OA was assessed by calculating OR with 95% confidence interval (95%CI). The stratification analysis was conducted in different ESR, age, gender, smoking, drinking, BMI, CRP. The adjusted ORs and their 95%CIs were also calculated, including age, gender, smoking, drinking, and BMI. We also used the Genotype Tissue Expression database to assess the correlation between SNP and ADAMTS14 (https://www.gtexportal.org/home/; Figure 1). All analyses were completed on SPSS 23.0, and *P*<0.05 was considered at significance level.

## Results

The general characteristics were presented in Table 1; 49.1% of OA patients were males and the ratio of control was 51.7%. The mean age of case group and control group were 57.1 and 56.6 years, respectively. The rates of smoking and drinking were 3.29 and 28.9% in the case group and 39.2 and 31.7% in the control group. The mean courses of disease were 1.2 for case group and 1.1 for control group. There were no significant differences in gender ratio (*P*=0.472), age (*P*=0.278), BMI (*P*=0.534), course of disease (*P*=0.056), smoking (*P*=0.067), and drinking (*P*=0.394). The positive rates of ACCP, AKA, APF, and RF were 73.1, 36.7, 44.5, and 16.8% in the case group. The ratios of ESR > 20 and CRP > 8 were 52.0 and 66.2%, respectively.

**Table 1:** Comparison of general characteristics between case and control groups

Factors	Case group (*n*=346)	Control group (*n*=480)	χ^2^/t	*P*
Gender			0.516	0.472
Male	170 (49.1%)	248 (51.7)		
Female	176 (50.9%)	232 (48.3)		
Age	57.1 ± 7.0	56.6 ± 7.0	−1.086	0.278
BMI	23.6 ± 3.0	23.8 ± 3.1	0.622	0.534
Course of disease	1.2 ± 0.8	1.1 ± 0.7	1.907	0.056
Smoking			3.352	0.067
Yes	114 (32.9%)	188 (39.2%)		
No	232 (67.1%)	292 (60.8%)		
Drinking			0.725	0.394
Yes	100 (28.9%)	152 (31.7%)		
No	246 (71.1%)	328 (68.3%)		
Kellgren–Lawrence grading		-		
I/II	206 (59.5%)			
III/IV	140 (40.5%)			
ESR ≥ 20	180 (52.0%)	-		
CRP > 8 mg/l	229 (66.2%)	-		
rs4747096			28.110	0.000
AA	146 (42.2%)	256 (53.3%)		
AG	126 (36.4%)	182 (37.9%)		
GG	74 (21.4%)	42 (8.8%)		

### Association of ADAMTS14 rs4747096 polymorphism with OA

Eight hundred and twenty-six samples were genotyped and the concordance rate of genotype was 100%. The distribution of genotype frequencies was 42.2% for AA, 36.4% for AG, and 21.4% for GG in the case group, and 53.3% for AA, 37.9% for AG, and 8.8% for GG. The Hardy–Weinberg equilibrium test indicated no significant differences in the case and in the control groups (*P*>0.05). Significant difference genotype differences were observed between case group and control group (χ^2^ = 28.110, *P*=0.000). The minor G allele in case group was significantly higher than in the control group (21.4 compared with 8.8%, *P*=0.000, OR = 1.71, 95%CI: 1.39–2.11). Significant differences in the distribution of genotypes were observed. The GG genotype and the GG/AG combination were more common in the OA group than in the control group. Compared with AA genotype, the GG (OR = 3.09, 95%CI: 2.01–4.75), AG (OR = 2.55, 95%CI: 1.64–3.96), and GG/AG (OR = 1.57, 95%CI: 1.19–2.07) increased the risk of OA. The codominant model also show the similar results (OR = 2.84, 95%CI: 1.89–4.27, *P*=0.000). We also conducted the multiple logistic to evaluate the relationship between rs4747096 and OA risk through adjusting some potential factors: age, gender, smoking, drinking, and BMI. The results were still significant in each model. The results were presented in Table 2.

**Table 2:** Logistic regression analysis of associations between ADAMTS14/rs4747096 gene polymorphism and KOA

Genotype	Cases		Control		OR (95% CI)	*P*	aOR (95%CI)	*aP*
	*n*	%	*n*	%				
GG compared with AA	74/146	21.4/42.2	42/256	8.8/53.3	3.09 (2.01–4.75)	0.000	3.26 (2.11–5.04)	0.000
GG compared with AG	74/126	21.4/36.4	42/182	8.8/37.9	2.55 (1.64–3.96)	0.000	2.57 (1.65–4.02)	0.000
GG/AG compared with AA	200/146	57.8/42.2	224/256	46.7/53.3	1.57 (1.19–2.07)	0.002	1.60 (1.21–2.12)	0.001
GG compared with AG/AA	74/272	21.4/78.6	42/438	8.8/91.2	2.84 (1.89–4.27)	0.000	2.98 (1.98–4.51)	0.000
G compared with A	274/418	39.6/60.4	266/694	27.7/72.3	1.71 (1.39–2.11)	0.000	-	-

Abbreviation: aP, adjusted P value; aOR, adjust OR: adjust age, gender, smoking, drinking, and BMI.

### Stratified analyses

We first conducted subgroup analyses in different age, gender, weight, smoking, and drinking. As shown in Table 3, significant differences were observed in AA compared with GG, AG compared with GG, AA/AG compared with GG, and A compared with G for male, and all models showed significant difference for female (*P*<0.05). For age, the models (AA compared with GG, AG compared with GG, A compared with G) showed significant difference (*P*<0.05). For smoking, there were significant differences between case group and control group. Patients with drinking and BMI ≥ 24 tend to show significant relationship between rs4747096 and OA. We further analyzed the relationship between these parameters and genotypes of rs4747096. There was no significant difference in allele frequency or genotypes frequency for these parameters in OA patients (*P*>0.05). Although the GG genotype was slightly higher in the CRP positive group than in CRP negative group, no significant difference was observed (*P*=0.376, Table 4).

**Table 3:** Subgroup analyses between ADAMTS14/rs4747096 gene polymorphism and the risk of KOA

Variables	rs4747096 (Case/control)			GG compared with AA		GG compared with AG		GG/AG compared with AA		GG compared with AG/AA		G compared with A	
	AA	AG	GG	OR (95%CI)	*P*	OR (95%CI)	*P*	OR (95%CI)	*P*	OR (95%CI)	*P*	OR (95%CI)	*P*
Sex													
Male	73/132	63/96	34/20	3.07 (1.65–5.73)	0.020	2.59 (1.37–4.89)	0.003	1.51 (1.02–2.24)	0.039	2.85 (1.58–2.15)	0.001	1.66 (1.24–2.23)	0.001
Female	73/124	63/86	40/22	3.09 (1.70–5.60)	0.000	2.48 (1.34–4.58)	0.004	1.62 (1.09–2.41)	0.017	2.80 (1.60–4.93)	0.000	1.76 (1.31–2.36)	0.000
Age (years)													
<60	78/156	82/118	50/28	3.57 (2.09–6.11)	0.000	2.57 (1.50–4.42)	0.001	1.28 (0.82–2.01)	0.277	3.05 (1.85–5.05)	0.000	1.89 (1.46–2.45)	0.000
≥60	68/100	40/64	24/14	2.52 (1.22–5.22)	0.013	2.49 (1.16–5.35)	0.019	1.81 (1.26–2.59)	0.001	2.51 (1.25–5.06)	0.010	1.43 (1.01–2.03)	0.042
Smoking													
Yes	42/108	41/60	31/10	2.78 (1.57–4.93)	0.000	2.81 (1.57–5.03)	0.001	2.31 (1.43–3.73)	0.001	2.79 (1.62–4.82)	0.000	2.89 (2.01–4.15)	0.000
No	104/148	85/122	43/22	3.99 (2.05–7.76)	0.000	2.27 (1.14–4.51)	0.020	1.26 (0.90–1.79)	0.183	3.14 (1.69–5.84)	0.000	1.47 (1.13–1.91)	0.004
Drinking													
Yes	40/80	40/52	20/10	4.08 (2.35–7.07)	0.000	3.71 (2.11–6.53)	0.000	1.53 (1.10–2.13)	0.012	3.91 (2.31–6.63)	0.000	1.77 (1.38–2.28)	0.000
No	106/176	86/130	54/22	2.00 (0.96–4.14)	0.062	1.30 (0.62–2.73)	0.490	1.67 (1.00–2.78)	0.050	1.65 (0.84–3.25)	0.148	1.47 (0.98–2.22)	0.063
BMI													
≥24	63/98	55/74	27/18	3.72 (2.13–6.52)	0.000	2.97 (1.68–5.30)	0.000	1.70 (1.18–2.45)	0.004	2.19 (1.15–4.15)	0.017	0.93 (0.66–1.30)	0.677
<24	83/158	71/108	47/48	2.33 (1.19–4.58)	0.014	2.02 (1.01–4.03)	0.046	1.39 (0.90–2.14)	0.140	3.38 (1.99–5.75)	0.000	1.18 (0.90–1.55)	0.219

**Table 4:** Comparison of genotypic and allelic frequencies of ADAMTS14/rs4747096 polymorphism amongst KOA subgroups stratified

Cases	GG	AG	AA	G	A
ESR ≥ 20	44 (24.4)	58 (32.2)	78 (43.3)	146 (40.6)	214 (59.4)
ESR < 20	30 (5.9)	68 (41.0)	68 (41.0)	128 (38.6)	204 (61.4)
*P*	0.395	0.224		0.591	
OR (95%CI)	1.28 (0.73–2.25)	0.74 (0.46–1.20)	1.00	1.09 (0.80–1.48)	1.00
CRP > 8 mg/l	54 (23.6)	77 (33.6)	98 (42.8)	185 (40.4)	273 (59.6)
CRP ≤ 8 mg/l	20 (17.1)	49 (41.9)	48 (41.0)	89 (38.0)	145 (62.0)
*P*	0.376	0.302		0.548	
OR (95%CI)	1.32 (0.71–2.45)	0.77 (0.47–1.27)	1.00	1.10 (0.80–1.53)	1.00
Grade I/II	33 (21.4)	52 (33.8)	69 (44.8)	118 (38.3)	190 (61.7)
Grade III/IV	41 (21.4)	74 (38.5)	66 (50.8)	156 (43.1)	206 (56.9)
*P*	0.708	0.322		0.209	
OR (95%CI)	0.90 (0.51–1.58)	0.78 (0.49–1.27)	1.00	0.82 (0.60–1.12)	1.00

## Discussion

It is well recognized that the susceptibility of KOA is influenced by genetic and environmental factors. In the past decades, a lot of studies had explored the associations between KOA and different candidate genes in different ethnic backgrounds [18,19]. However, results of most susceptibility genes identified have not been replicated in subpopulations different from population used in the previous study. Poonpet et al. [20] found the SNP rs4747096 in ADAMTS14 was associated with KOA in female Thai patients. After that, studies about association between ADAMTS14 and OA are few. To our best knowledge, this is the first study about association between ADAMTS14 and KOA in Chinese population, including males and females. Our study with more sample size indicated that the ADAMTS14 rs4747096 gene polymorphism was associated with KOA in Chinese Han population.

One of the primary pathology symptoms of OA is the degeneration of articular cartilage, characterized by degradation and synthesis imbalance of chondrocytes, extracellular matrix, and subchondral bone that was usually caused by mechanical and biological factors. The articular cartilage of the knee is fibrous cartilage, and collagen in the cartilage matrix accounts for 50–60% (mainly type II collagen) [21]. The type II collagen is essential to maintain the mechanical strength of cartilage because the collagen can hold on the stress of cartilage. The destruction of collagen fibers in superficial articular cartilage is an important histopathological signal of early stage OA. The experiments *in vivo* have suggested that the diameter of collagen fibers in early-stage OA articular cartilage increases, and the changes in the microstructure of usually occur earlier than subchondral bone destruction [22].

ADAMTS is a group of secretory proteases with the metalloproteinase domain, depolymerization domain, and platelet reactive protein domain, which is involved in the degradation of collagen polysaccharide in cartilage matrix. Studies on gene polymorphism of ADAMTS family member have become the focus of the pathogenesis of OA. Li et al. [23] found that the serum ADAMTSs level in the early KOA was significantly higher than that in the healthy people. However, the only serum ADAMTS5 level of advanced patients were significantly higher than that in the healthy people and speculated that the ADAMTS4 might be a serological marker of early KOA [23]. Kumar et al. [24] found that the OA severity mice with ADAMTS4/5 knockout was more significantly alleviated than that in the wild-type. Miller et al. [25] reported that the application of ADAMTS5 antibody could slow down the cartilage damage in mice with KOA. Gok et al. [26] reported that patients with >20 repeated sequences in *ADAMTS9* gene had more serious KOA. The ADAMTS1/4/5/8/9/15 can promote the degradation of proteoglycan in articular cartilage while ADAMTS7/9/15 were involved in the degradation of oligomeric proteins in cartilage matrix [26]. These results indicated that *ADAMTS* gene was associated with OA.

*ADAMTS14* gene is located on chromosome 10q222.1. At present, the function of ADAMTS14 is not fully clear. Since *ADAMTS14* and *ADAMTS3* genes (63%) are homologous, Fernandes et al. [27] speculated that ADAMTS14 and ADAMTS3 have similar functions, which can crack the N-terminal anterior peptide of collagen monomer, promote the aggregation of type II collagen monomer into type II collagen fiber, and participate in the repair process after cartilage damage. The protein encoded by *ADAMTS14* gene consists of 1223 amino acids, which are composed of signal peptide, restructure domain, protease structural domain, poly structural domain, four thrombin-sensitive protein structural domains, and carboxyl terminal structural domain. The four thrombin-sensitive protein domains were separated by cysteine-rich amino acid fragments. The rs4747096 (A>G) G allele is the ancestral allele, but A allele accounts for larger frequency in European/Asian/African populations [28]. So G allele may be the dominant gene in the natural selection process. Changes in the sequence of bases result in the conversion of amino acids from glutamate (GAA) into GGA in the carboxyl terminal structure of the encoded protein molecule. We hypothesized that the polymorphism of ADAMTS14 gene rs747096 may cause the abnormal maturation process of type II collagen and lead to cartilage destruction, but this process still needs further research and verification. Results from GETs database indicated the GG genotype had lower level of ADAMTS gene expression, which suggested that the mutant gene cannot be expressed. The GG genotype made the ADAMTS14 expression level decreased. This results further indicated the close relationship existed between ADAMTS14 and KOA. We compared the genotype frequency of ADAMTS14 rs4747069 in Chinese Han population and found there was significant difference in genotype frequency between KOA patients and control group. The GG genotype frequency in the case group was significantly higher than that in the control group. Compared the control, the case have higher risk of KOA (OA = 3.09, 95%CI: 2.01–4.75). This result was different from previous two studies. Previous studies reported that the AA genotype was a risk gene. Poonpet et al. [20] reported a nearly significant association was found between the AG genotype and KOA in female patients (OR = 2.65; 95% CI = 0.99-7.19; P = 0.031). In contrast, the rs4747096 polymorphism was not associated with OA susceptibility in males in the present study. This result is doubtful. The upper CI is almost equal to 1. The significance was very weak. The sample size was so small in their study. We calculated the power of the present study and it is only 64.7% in the dominance model (significance model in female). The power of our study is more than 90% in the same model of inheritance [28]. Similarly, Wang et al. [29] investigate the association between SNPs of ADAMTS14 gene rs474096 and OA of the temporomandibular joint in Chinese Han females, and a quiet weak association was found (OR = 1.114, 95%CI:1.015-1.223) in the dominance model, and the power of the present study was 58.8%. More importantly, both of the studies did not adjust the confounding factors. Considering the sample size, power, and confounding factors, we believe previous studies may be false positive association. Our study results with adjusted several potential confounding factors give more strong evidence. A study with a variety of phenotypes totaling 3217 OA patients and 2214 healthy controls reported that the rare allele of the rs4747096 nsSNP in ADAMTS14 was overrepresented in women requiring joint replacement because of KOA (OR = 1.41, 95%CI = 1.1–1.8; *P*=0.002) and in patients with symptomatic hand OA (OR = 1.37, 95%CI = 1.0–1.9; *P*=0.047). The trend results of the present study was consistent with our reports [30]. Our results indicated that the rare allele gene increased the risk of KOA.

Our study has several limitations. A major limitation to the present study is that this is a case–control study design. This point could be found in all studies between gene polymorphism and diseases. This limitation might be overcome by the further exploring the mechanism. The other limitation is that some potential cofounding factors might be not included in the present study, which may overestimate or underestimate the effect of gene polymorphism. Further study is required.

In conclusion, our results indicated that ADAMTS14 gene polymorphism was associated with KOA, and the GG genotype of rs4747096 increased the risk of KOA in Chinese Han population. This is the first study to demonstrate that a correlation exists between ADAMTS14 gene polymorphisms and KOA. The ADAMTS14 may be a diagnostic marker and therapeutic target for KOA treatment. The future study should explore the specific molecular mechanism.

## Abbreviations

ACCP: anti-cyclic citrullinated peptide antibody
ADAMTS: A disintegrin and metalloproteinase with thrombospondin motifs
APF: antiperinuclear factor
BMI: body mass index
CI: confidence interval
CRP: C-reactive protein
KOA: knee osteoarthritis
OA: osteoarthritis
OR: odds ratio
SNP: single nucleotide polymorphism
